# Microtubules regulate cardiomyocyte transversal Young’s modulus

**DOI:** 10.1073/pnas.1917171117

**Published:** 2020-01-27

**Authors:** Pamela Swiatlowska, Jose L. Sanchez-Alonso, Peter T. Wright, Pavel Novak, Julia Gorelik

**Affiliations:** ^a^National Heart and Lung Institute, Imperial College London, W12 0NN London, United Kingdom;; ^b^School of Engineering and Materials Science, Queen Mary University of London, E1 4NS London, United Kingdom

**Keywords:** mechanobiology, heart failure, microtubules, Young’s modulus

## Abstract

The field of cardiomyocyte mechanobiology is gaining significant attention, due to accumulating evidence concerning the significant role of cellular mechanical effects on the integrated function of the heart. To date, the protein titin has been demonstrated as a major contributor to the cardiomyocytes Young’s modulus (YM). The microtubular network represents another potential regulator of cardiac mechanics. However, the contribution of microtubules (MTs) to the membrane YM is still understudied and has not been interrogated in the context of myocardial infarction (MI) or mechanical loading and unloading. Using nanoscale mechanoscanning ion conductance microscopy, we demonstrate that MTs contribute to cardiomyocyte transverse YM in healthy and pathological states with different mechanical loading. Specifically, we show that posttranslational modifications of MTs have differing effects on cardiomyocyte YM: Acetylation provides flexibility, whereas detyrosination imparts rigidity. Further studies demonstrate that there is no correlation between the total protein amount of acetylated and detyrosinated MT. Yet, in the polymerized-only populations, an increased level of acetylation results in a decline of detyrosinated MTs in an MI model.

The mechanical environment of a cell contributes to its development, differentiation, proliferation, and apoptosis. Mechanical effects are also intrinsic to the pathophysiological processes of disease onset and progression (1). The heart is particularly interesting for its dynamic contractile cycle. Titin and collagen have been identified as regulators of cardiac Young’s modulus (YM), a characteristic property of cell mechanobiome (2). Nonetheless, other potential contributors may have been overlooked. The cellular microtubules (MTs) are major constituents of the cytoskeleton and have been proposed as an important regulator of cardiac mechanics. However, data published over the last 30 years concerning the role of MTs in the cellular mechanobiome have been contradictory (3–7). Long-lived MTs are targeted by several posttranslational modifications (PTMs), such as acetylation and detyrosination (8, 9), which may affect their mechanical properties. A number of novel techniques have been introduced in the past few years to map cell mechanics; however, they all have technical limitations when working with live cells (10–12). In this study, we used noncontact, nanoscale mechanoscanning ion conductance microscopy (mechanoSICM) to map surface membrane domains (z-groove and crest) and confirm that MTs contribute to cardiomyocyte transverse YM. We also interrogated the MT contribution to YM upon myocardial infarction as well as upon mechanical unloading and study the correlation between PTMs.

## Results and Discussion

**Fig. 1.**
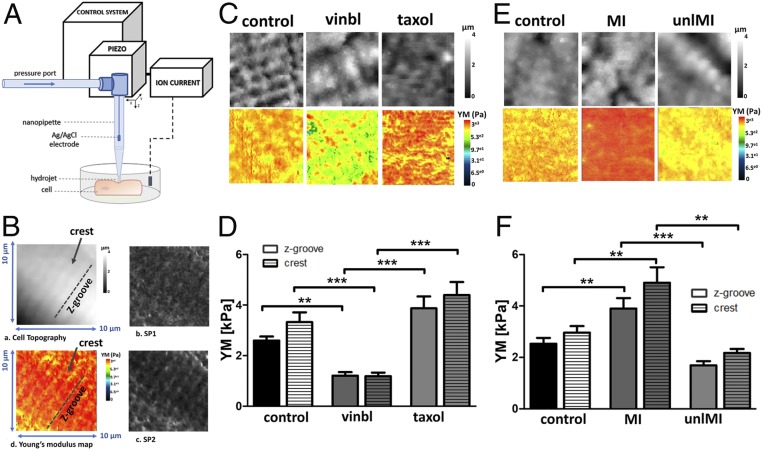
MTs regulate cardiomyocyte transverse YM as measured by mechanoSICM. (*A*) MechanoSICM schematic. (*B*) Representative cardiomyocyte topography image outlining crests and z-grooves (*a*), setpoint 1 (SP1) image taken at 1% decrease in current (*b*), setpoint 2 (SP2) image taken at 2% decrease in current (*c*), and example of YM map generated from the same area (*d*). (*C* and *E*) Representative SICM images of topography and YM maps. (*D*) Quantification of YM in control, vinblastine (vinbl), and taxol, *n* = 21 to 24 cells, three to five isolations. (*F*) Quantification of YM in control, MI, and unlMI, *n* = 20 to 66 cells, three to six isolations. **P < 0.01, ***P < 0.001.

**Fig. 2.**
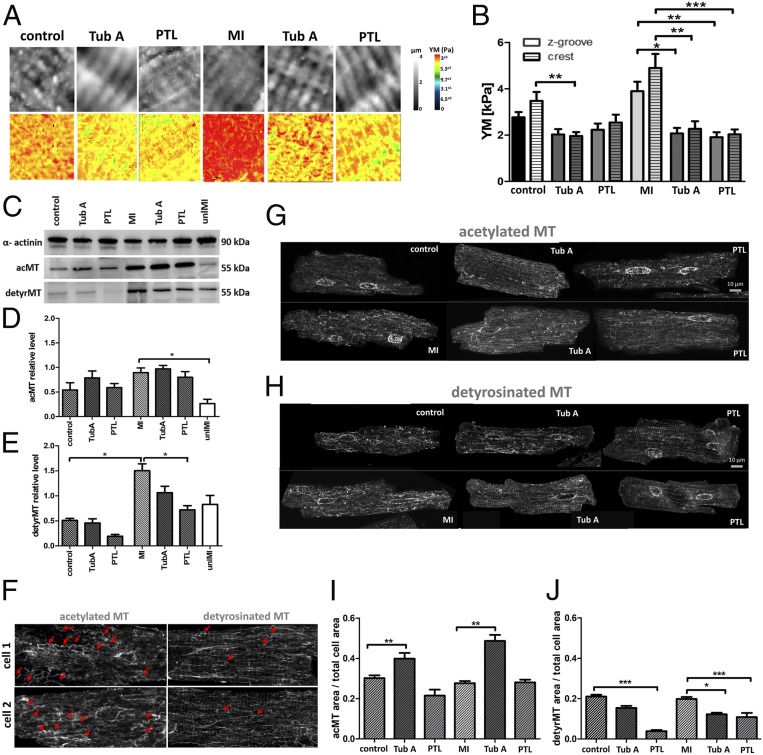
MT PTMs play a major role in regulating cardiomyocyte YM. Representative SICM images of topography and YM maps (*A*). Quantification of YM (*B*); *n* = 15 to 34 cells, three to five isolations. WB (*C*) and representative images of immunofluorescence micrographs (*G* and *H*). Quantitative analysis of the WB normalized to α-actinin, *n* = 4 to 6 isolations (*D* and *E*) and immunofluorescent staining, *n* = 13 to 26 cells, three to four isolations (*I* and *J*). Representative micrographs of curved MT indicated by red arrows (*F*). *P < 0.05, **P < 0.01, ***P < 0.001.

MechanoSICM is a noncontact method that simultaneously generates topographical and YM maps of the cell membrane. A constant pressure is applied from the pipette tip during scanning, which exerts a mechanical force on the cell membrane (Fig. 1 *A* and *B*). This will test the YM values in cell membrane nanodomains z-grooves and sarcolemma crests. First, we treated cells with vinblastine, an MT depolymerizing agent, or taxol, an MT stabilizing compound, to observe their effects on the cardiomyocytes YM. We observed a clear correlation between the integrity of MT and cellular YM. There was a significant drop in the YM of vinblastine-treated cells (control z-groove: 2.59 ± 0.16 kPa, crest: 3.32 ± 0.38 kPa vs. vinblastine z-groove: 1.2 ± 0.14 kPa, crest: 1.18 ± 0.14 kPa, *P* < 0.01) and an increase in YM of taxol-treated cells (taxol z-groove: 3.87 ± 0.47 kPa, crest: 4.39 ± 0.51 kPa) in both nanodomains (Fig. 1 *C* and *D*). Second, we used a rat model of MI to study how the pathological increase in workload modifies YM and MT content. We also studied cardiomyocytes from a partial mechanical unloading myocardial infarction (MI) model (unlMI). Consistent with previous reports, we observed an increased YM in cells derived from the MI model (control z-groove: 2.5 ± 0.22 kPa, crest: 2.96 ± 0.25 kPa vs. MI z-groove: 3.89 ± 0.4 kPa, crest: 4.9 ± 0.6 kPa, *P* < 0.01) (Fig. 1 *E* and *F*), together with a densification of detyrosinated and acetylated MTs (detyrMT control: 0.51 ± 0.03 vs. MI: 1.5 ± 0.15, *P* < 0.05; acMT control: 0.54 ± 0.14 vs. MI: 0.89 ± 0.09, *P* > 0.05) (Fig. 2 *C*–*E*). YM in the crests is higher than in the z-grooves in all groups, although the difference is not statistically significant. In unlMI cells, YM is lower (unlMI z-groove: 1.68 ± 0.16 kPa, crest: 2.17 ± 0.15 kPa vs. MI, *P* < 0.01) (Fig. 1 *E* and *F*), and MT acetylation is significantly decreased (unlMI: 0.26 ± 0.08 vs. MI, *P* < 0.05), together with a reduction in detyrosination levels (unlMI: 0.82 ± 0.17 vs. MI) (Fig. 2 *C*–*E*). This suggests that mechanical unloading could mitigate the effects of MI on cell YM. Robison et al. (8) reported that genetic or pharmacological suppression of MT detyrosination induces cell softening. We incubated control and MI cardiomyocytes with parthenolide (PTL, detyrosination inhibitor) and found that cells became softer, significantly in the MI group (PTL z-groove: 2.23 ± 0.23 kPa, crest: 2.54 ± 0.34 kPa vs. control; MI PTL z-groove: 1.91 ± 0.21 kPa, crest: 2.03 ± 0.2 kPa vs. MI, *P* < 0.01) (Fig. 2 *A* and *B*). Next, we treated control and MI cells with tubastatin A (TubA), which introduces more acetyl groups to MTs. This treatment also results in cell softening (TubA z-groove: 2.03 ± 0.23 kPa, crest: 1.96 ± 0.17 kPa vs. control, *P* < 0.01 for crest; MI TubA z-groove: 2.07 kPa ± 0.23 kPa, crest: 2.27 ± 0.32 kPa vs. MI, *P* < 0.01) (Fig. 2 *A* and *B*). Next, we investigated if this softening effect is due to interactions between acMT and detyrMT populations. Using Western blotting (WB) we observed a decreased level of detyrMT after PTL treatment (*P* = 0.058) in the control group and a significant reduction in the MI group (*P* < 0.05). Following TubA treatment, acMT levels were slightly increased, but detyrMT levels were reduced (Fig. 2 *C*–*E*). Likewise, in the MI cells, with increased acetylation by TubA, the density of detyrMT, as quantified on the immunofluorescent micrographs, decreased significantly (Fig. 2 *H* and *J*). No change in the acetylation level was observed when detyrMT were down-regulated (Fig. 2 *C*–*E*, *G*, and *I*), suggesting that the softening effect of increasing acetylation through TubA could be caused in part by the reduction in detyrMT, which has been clearly related to cardiomyocytes mechanics (8). In fact, increased stiffness of the MI cells could also be related to a higher expression of detyrMT, as seen by WB (Fig. 2*E*), whereas no significant increase is observed in the alpha-tubulin (control: 0.26 ± 0.07 vs. MI: 0.39 ± 0.12, *n* = 5 isolations). We note a difference in our WB findings with respect to studies with immunostaining. Our preferred explanation for this discrepancy is that WB studies include thousands of cells, whereas the immunostaining analysis assessed a limited population. We have also noticed in the studies using immunostaining some regional variations in the MT network density (surface vs. center), but to clarify this will require an extensive study.

Using mechanoSICM we provide evidence that MTs can contribute to cardiomyocyte transverse YM and tune the mechanical properties. We observe that the stiffer detyrMT are accompanied by more flexible acMT that tend to have a more curved organization than detyrMT (Fig. 2*F*), suggesting a protective role for the load-bearing detyrMT. The precise nature of the cross-talk between these two PTMs remains poorly defined, but a potential mechanism could involve members of the kinesin family (13). In fact, MT acetylation promotes kinesin-1 binding, whereas an increase in kinesin-1 inhibits MT detyrosination (14, 15). The involvement of other PTMs like polyglutamylation could also be considered for future studies as they could be playing a role in MT mechanics.

This work also shows how different cardiac loading conditions can change MT PTMs. In agreement with other reports, we show an MT network densification in the MI models. We also show that in unlMI there is a decrease in the pathological levels of PTMs. This study shows that MT contribution to cell YM is reversible upon unloading. The unlMI mimics several features of left ventricular assist devices in patients (16). This therapy has been questioned in some cases as prolonged unloading can attenuate cardiac function (17). Here we observe that unloading the heart is beneficial for the MT network, but future studies may reveal other critical factors responsible for the outcomes observed in patients.

## Materials and Methods

### Models.

All animal experiments were approved by the Animal Welfare and Ethical Review Board (AWERB) of Imperial College London, UK and carried out in accordance with the UK Animals (Scientific Procedures) Act 1986, incorporating the European Union Directive 2010/63/EU.

Age-matched male Sprague-Dawley rats were used for control and MI models, generated according to previously published studies (18). Age-matched male Lewis rats were used for the MI and partial unlMI studies, with respective controls (16). MI experiments were performed 16 wk after coronary ligation, when end-stage heart failure (HF) develops. To assess the effect of unloading on failing hearts, heterotopic transplantations were performed at 16 wk and the hearts were maintained for a further 8 wk when experiments were performed (unlMI). This allows us to assess the mechanisms involved in the therapeutic effect of unloading at end-stage HF.

Cell isolation was achieved by Langendorf perfusion as described in detail in previous work (19). Isolated cardiomyocytes were plated on laminin-coated dishes. As required, cells were incubated with vinblastine (1 h, 1 µM), taxol (2 h, 10 µM), parthenolide (2 h, 10 µM), or TubA (3 h, 2 µM) at 37 °C, 5% CO_2_. Treated cells were compared to respective controls in the same conditions. Reagents were diluted in dimethyl sulfoxide (DMSO) except for vinblastine, which was diluted in water, when applied DMSO concentration in the bath was 0.1 to 0.01%.

### YM Measurement by SICM.

To map YM, cells were scanned using a modification of SICM in “hopping” mode (mechanoSICM) (19). Aerostatic, constant pressure (15 kPa) was delivered to the nanopipette (250- to 350-nm diameter) and propelled the inner solution, forming a hydrojet, applied throughout the scanning procedure (10 µm × 10 µm). The current–distance dependence was used to map the topography (0.7% of current reduction) and YM maps with two additional channels, SP1 and SP2, 1% and 2% current reduction, respectively (Fig. 1*B*). Topographical slope correction was introduced to correct for the uneven membrane surface. Between three and five measurements in each domain (z-groove and crest) were averaged per cell.

### WB.

Cells were lysed using RIPA buffer and proteinase inhibitors were added. Protein concentration was determined using bicinchoninic acid assay. Samples were resolved on 10% sodium dodecyl sulfate polyacrylamide gels and overnight transfer was performed at 4 °C. Membranes were blocked with 5% milk, diluted in Tris-buffered saline with Tween 20 (TBST), and incubated overnight with primary antibodies acetylated tubulin (1:1,000, sc-23950; Santa Cruz Biotechnology), detyrosinated tubulin (1:600, ab3201; Abcam), and α-actinin (1:000, A7811; Sigma-Aldrich). Finally, membranes were washed three times with TBST and incubated with secondary horseradish peroxidase-linked antibody for 1 h.

### Immunofluorescence.

Cells were fixed with 4% formaldehyde or ice-cold methanol and formaldehyde for 15 min, blocked for 1 h with 1% bovine serum albumin + 0.1% Tween, and stained with acetylated and detyrosinated tubulin primary antibodies for 2 h. Samples were incubated with secondary antibodies for 1 h (Alexa Fluor 488). Z-stack images were quantified by the intensity thresholding and standardized to the respective cell size using ImageJ. Five slices from the z-stack were averaged per each cell, two from the submembrane region and three from the cell center.

### Statistics.

All datasets were tested for the normal distribution before ANOVA and post hoc tests were carried out. When the data fail the normality test nonparametric Kruskal–Wallis test was used. Data are presented as ± SEM; *P < 0.05, **P < 0.01, ***P < 0.001.

### Data Availability.

Data are available upon request to the corresponding author.
